# SLMP53-1 Inhibits Tumor Cell Growth through Regulation of Glucose Metabolism and Angiogenesis in a P53-Dependent Manner

**DOI:** 10.3390/ijms21020596

**Published:** 2020-01-17

**Authors:** Helena Ramos, Juliana Calheiros, Joana Almeida, Valentina Barcherini, Sónia Santos, Alexandra T. P. Carvalho, Maria M.M. Santos, Lucília Saraiva

**Affiliations:** 1 LAQV/REQUIMTE, Laboratόrio de Microbiologia, Departamento de Ciências Biolόgicas, Faculdade de Farmácia, Universidade do Porto, 4050-313 Porto, Portugal; helenainrr@gmail.com (H.R.); julianameixedo@ua.pt (J.C.); JoanaAlmeida15@gmail.com (J.A.); 2 Research Institute for Medicines (iMed.ULisboa), Faculty of Pharmacy, Universidade de Lisboa, 1649-003 Lisboa, Portugal; vbarcherini@ff.ulisboa.pt (V.B.);; 3 CNC—Center for Neuroscience and Cell Biology, Institute for Interdisciplinary Research (IIIUC), University of Coimbra, 3004-504 Coimbra, Portugal; sonia.gomes.santos@hotmail.com (S.S.); atpcarvalho@uc.pt (A.T.P.C.)

**Keywords:** p53, anticancer drug, glycolysis, OXPHOS, anti-angiogenic, anti-migratory

## Abstract

The Warburg effect is an emerging hallmark of cancer, which has the tumor suppressor p53 as its major regulator. Herein, we unveiled that p53 activation by (*S*)-tryptophanol-derived oxazoloisoindolinone (SLMP53-1) mediated the reprograming of glucose metabolism in cancer cells and xenograft human tumor tissue, interfering with angiogenesis and migration. Particularly, we showed that SLMP53-1 regulated glycolysis by downregulating glucose transporter 1 (GLUT1), hexokinase-2 (HK2), and phosphofructokinase-2 isoform 6-phosphofructo-2-kinase/fructose-2,6-biphosphatase-3 (PFKFB3) (key glycolytic enzymes), while upregulating the mitochondrial markers synthesis of cytochrome *c* oxidase 2 (SCO2), cytochrome *c* oxidase subunit 4 (COX4), and OXPHOS mitochondrial complexes. SLMP53-1 also downregulated the monocarboxylate transporter 4 (MCT4), causing the subsequent reduction of lactate export by cancer cells. Besides the acidification of the extracellular environment, SLMP53-1 further increased E-cadherin and reduced metalloproteinase-9 (MMP-9) expression levels in both cancer cells and xenograft human tumor tissue, which suggested the interference of SLMP53-1 in extracellular matrix remodeling and epithelial-to-mesenchymal transition. Consistently, SLMP53-1 depleted angiogenesis, decreasing endothelial cell tube formation and vascular endothelial growth factor (VEGF) expression levels. SLMP53-1 also exhibited synergistic growth inhibitory activity in combination with the metabolic modulator dichloroacetic acid. These data reinforce the promising application of the p53-activating agent SLMP53-1 in cancer therapy, by targeting p53-mediated pathways of growth and dissemination.

## 1. Introduction

The reprogramming of energy metabolism recently emerged as a new hallmark of cancer [1]. In particular, a common property of cancer cells is an altered glucose metabolism compared to normal cells. In fact, even in the presence of sufficient oxygen, most cancer cells increased glucose consumption and converted it to lactate, instead of relying on mitochondrial oxidative phosphorylation (OXPHOS), a phenomenon termed the “Warburg effect” [1,2]. Glycolysis is a faster but less efficient process than OXPHOS in ATP production, but this inefficiency can be compensated by increasing glucose uptake through the upregulation of transmembrane glucose transporters (GLUT) [3]. In fact, the Warburg effect confers several advantages to cancer cells, particularly by providing intermediates for different biosynthetic pathways required for unrestrained proliferation, and an adaptation to hypoxic conditions often observed in solid tumors [4]. Moreover, increased glycolysis benefits not only the tumor growth, but also its dissemination. The spreading of cancer cells in a multicellular environment is a direct consequence of the elevated glucose consumption and subsequent lactate secretion, which decreases the pH of the microenvironment. An acidic environment leads to the death of surrounding normal cells and extracellular matrix (ECM) remodeling by proteolytic enzymes, including metalloproteinases (MMP) [4,5], and depletion of cell-cell adhesion proteins, such as E-cadherin (E-CAD) [6]. This promotes epithelial-to-mesenchymal transition (EMT) [7,8], which enables cancer cells to migrate and invade. Low pH also promotes angiogenesis through the enhanced expression of the vascular endothelial growth factor (VEGF) by cancer [9] and endothelial [10] cells. Additionally, lactate activates the transcriptional hypoxia-inducible factor (HIF), which was shown to regulate a myriad of genes involved in angiogenesis [11] and metastasis [12] processes. Another consequence of the Warburg effect is the decreased usage of the mitochondrial respiratory chain, due to reduced OXPHOS and oxygen consumption. Accordingly, less reactive oxygen species (ROS) are produced, which promotes cancer-cell proliferation and apoptosis evasion [13].

Metabolic reprogramming in cancer cells is driven by activation of oncogenes (e.g., *c*-Myc and HIF) and/or inactivation of tumor suppressors, including p53 [14,15]. Numerous studies have shown that p53 intervenes at many steps in the glucose metabolism by slowing down glycolysis and promoting OXPHOS (reviewed in [16]). In particular, p53 downregulates GLUT1/4 [17] and GLUT3 (through inhibition of factor nuclear kappa B, NF-κB), limiting glucose import [18]. It also transcriptionally downregulates hexokinase 2 (HK2), which phosphorylates glucose to produce glucose-6-phosphate (G6P) as the first step of glucose metabolism [19], and participates in phosphoglycerate mutase (PGM) degradation routes [16], compromising glycolysis. Furthermore, in response to DNA damage, p53 promotes nucleotide biosynthesis by repressing the expression of the phosphofructokinase-2 isoform 6-phosphofructo-2-kinase/fructose-2,6-biphosphatase 3 (PFKFB3), a rate-limiting enzyme that promotes glycolysis [20]. On the other hand, p53 induces the expression of synthesis of cytochrome *c* oxidase 2 (SCO2), which assists in the assembly of mitochondrial complex IV of the mitochondrial electron transport chain [21], as well as of apoptosis-inducing factor (AIF), which is required for efficient OXPHOS, ensuring the proper assembly and function of mitochondrial respiratory complex I [22]. As such, p53 activation has been regarded as a promising targeted therapeutic approach to reprogram tumor glucose metabolism, conducting cancer cell death [16,23].

Recently, our group reported the (*S*)-tryptophanol-derived oxazoloisoindolinone (SLMP53-1) as an activator of wild-type (wt) and mutant (mut)p53 with promising application in cancer therapy [24]. SLMP53-1 binds to p53, enhancing its DNA-binding ability and subsequent transcriptional activity [24]. Additionally, it displays *in vitro* and *in vivo* p53-dependent antitumor activity, with no apparent undesirable toxicity [24]. Key physicochemical and pharmacokinetic parameters of SLMP53-1, calculated by using SwissADME [25], also showed that SLMP53-1 obeyed criteria for drug-likeness, gastrointestinal adsorption, lipophilicity, and solubility (Supplementary Tables S1–S7). These data prompted us to further investigate the potential of SLMP53-1 as an anticancer drug candidate.

Herein, we carried out an in-depth analysis of the molecular events underlying the antitumor activity of SLMP53-1 by studying its effect on glucose metabolism and angiogenesis.

## 2. Results

### 2.1. SLMP53-1 Regulates the Warburg Effect and Angiogenesis in Cancer Cells, with Interference in ECM Remodeling and EMT Events

**Figure 1:**
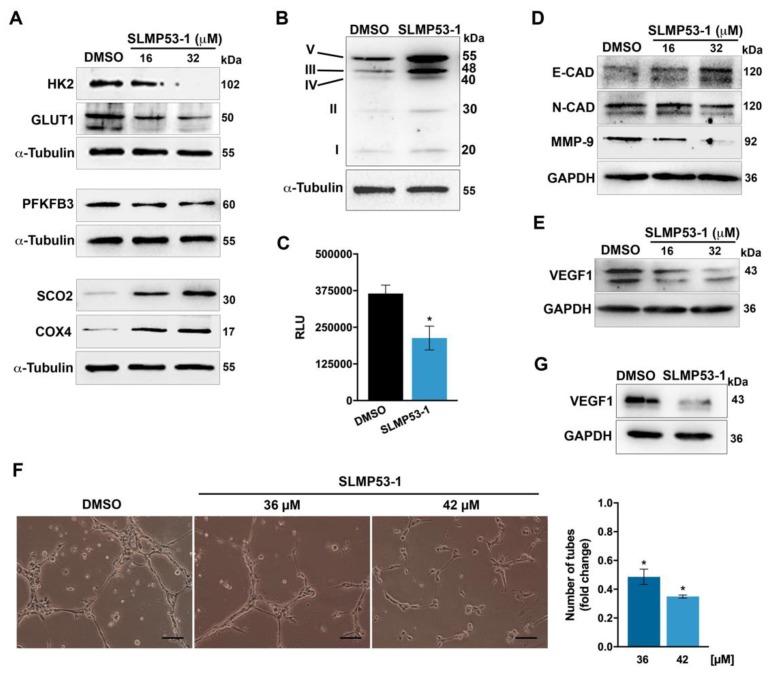
SLMP53-1 modulates the Warburg effect and angiogenesis in cancer cells, with impact on ECM remodeling and EMT events in vitro. (**A**) Expression levels of proteins involved in glycolysis and OXPHOS after 24 h treatment with SLMP53-1, in HCT116 cancer cells. (**B**) Expression levels of OXPHOS mitochondrial complexes I–V, after 24 h treatment with 16 µM SLMP53-1, in HCT116 cells. (**C**) Effect of 16 µM SLMP53-1 on lactate secretion by HCT116 cells, after 24 h treatment. Relative luminescence units (RLU) signal was normalized to the respective cell number and corresponds to mean ± SEM of three independent experiments. Values significantly different from DMSO (* *p* < 0.05; unpaired Student’s *t*-test). (**D**,**E**) Expression levels of proteins involved in migration (**D**) and angiogenesis (**E**) after 24 h treatment with SLMP53-1, in HCT116 cells. (**F**) Anti-angiogenic effect of SLMP53-1, in HMVEC-D cells, after 12 h treatment, using the endothelial cell tube formation assay. Representative images are shown (scale bar = 50 μm and magnification = ×100; zoomed-out pictures are shown in Supplementary Figure S1A). Quantification of tube-like structures in five randomly selected microscopic fields; fold changes are relative to solvent (DMSO) and correspond to mean ± SEM of three independent experiments. Values significantly different from DMSO (* *p* < 0.05; one-way ANOVA with Dunnett’s multiple comparison test); quantification of total tube length, using Image J software, is shown in Supplementary Figure S1B. (**G**) Expression levels of VEGF1, after 48 h treatment with 42 µM SLMP53-1, in HMVEC-D cells. In (**A**,**B**,**D**,**E**,**G**), immunoblots are representative of three independent experiments; α-tubulin or GAPDH were used as a loading control.

To investigate whether SLMP53-1 could interfere with the Warburg effect, the expression levels of major proteins involved in glycolysis and OXPHOS were investigated in HCT116 cancer cells. When compared to solvent, SLMP53-1 downregulated the protein levels of GLUT1, HK2, and PFKFB3, while it upregulated the mitochondrial markers SCO2 and cytochrome *c* oxidase subunit 4 (COX4), particularly for 32 µM (Figure 1A). Moreover, 16 µM SLMP53-1 upregulated the levels of OXPHOS mitochondrial complexes, with a more pronounced effect on complexes III and V (Figure 1B). These results indicated the modulation of the Warburg effect by SLMP53-1, with glycolysis inhibition and OXPHOS stimulation, in HCT116 cells. The anti-glycolytic activity of SLMP53-1 was further sustained by the reduction of extracellular lactate levels in SLMP53-1-treated HCT116 cells, when compared to solvent (Figure 1C).

In HCT116 cells, it was also observed that SLMP53-1, particularly at 32 µM, increased the protein levels of E-CAD, while decreasing the levels of N-cadherin (N-CAD) and MMP-9 (Figure 1D). These data were consistent with an inhibition of ECM remodeling and EMT processes.

We next checked the anti-angiogenic potential of SLMP53-1. To this end, we started by assessing the effect of SLMP53-1 on VEGF1 expression levels, in HCT116 cells. The results showed that 16 and 32 µM SLMP53-1 visibly decreased the VEGF1 protein levels (Figure 1E). In addition, we tested the effect of SLMP53-1 on endothelial cell tube formation. For that, the anti-proliferative effect of SLMP53-1 on HMVEC-D endothelial cells was previously determined. An IC_50_ (half maximal inhibitory concentration) value of 74 ± 10.2 μM (three independent experiments; 48 h treatment) indicated low toxicity of SLMP53-1 toward endothelial cells. Thereafter, using the endothelial cell tube formation assay, a pronounced anti-angiogenic effect of SLMP53-1 could be observed. In fact, 36 and 42 μM of SLMP53-1 led to a significant decrease in HMVEC-D tube formation, upon 12 h treatment (Figure 1F). Notably, for these concentrations and treatment time, SLMP53-1 had no significant cytotoxic effect on HMVEC-D cells. Consistently, 42 µM SLMP53-1 also decreased the VEGF1 protein levels in HMVEC-D cells (Figure 1G).

### 2.2. SLMP53-1 Regulates the Warburg Effect and Angiogenesis, Interfering with ECM Remodeling and EMT Events, in a p53-Dependent Manner, in Tumor Tissues of Xenograft Mouse Models

**Figure 2:**
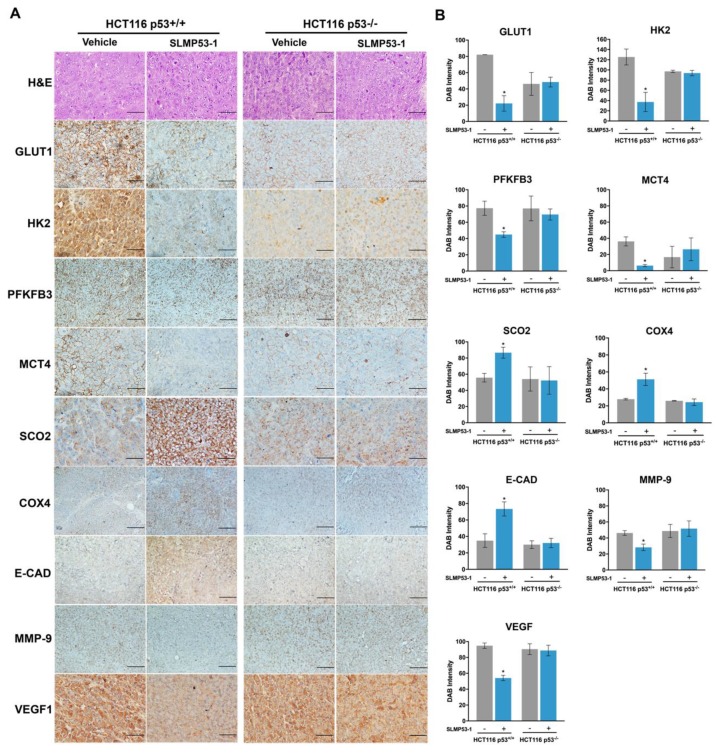
SLMP53-1 modulates the Warburg effect and angiogenesis, interfering with ECM remodeling and EMT events, through a p53-dependent pathway, in tumor tissues of xenograft mouse models. (**A**,**B**) Detection of molecular markers involved in glycolysis, OXPHOS, angiogenesis, and migration, in tumor tissues of p53^+/+^ and p53^−/−^ HCT116 xenografts treated with 50 mg/kg SLMP53-1 or vehicle (intraperitoneal injection twice a week, for a total of five administration [24]); in (**A**) representative images of IHC are shown; scale bar =20 μm; magnification = ×200; hematoxylin and eosin (H&E); in (**B**) quantification of IHC staining in tumor tissues of p53^+/+^ and p53^−/−^ HCT116 xenografts treated with SLMP53-1 (+) or vehicle (−). Staining quantification was assessed by evaluation of DAB (3,3′-diaminobenzidine) intensity. Data are mean ± SEM, values significantly different from vehicle (* *p* < 0.05, unpaired Student’s *t*-test).

To assess the in vivo effect of SLMP53-1 on glucose metabolism, the expression levels of glycolytic and OXPHOS molecular markers were evaluated by immunohistochemistry (IHC) staining in tumor tissues of xenograft mouse models carrying p53^+/+^ and p53^−/−^ HCT116 cells obtained in previous work [24] (Figure 2A,B). The results showed that SLMP53-1 treatment (50 mg/kg, twice a week during two weeks) modulated the protein levels of typical glycolytic and OXPHOS markers, in p53-expressing, but not in p53-null, tumor tissues. In fact, when compared to vehicle, SLMP53-1 treatment decreased the expression levels of GLUT1, HK2, monocarboxylate transporter 4 (MCT4), and PFKFB3 in p53^+/+^, but not in p53^−/−^, HCT116 tumors (Figure 2A,B). Conversely, SLMP53-1 promoted OXPHOS by enhancing the expression of SCO2 and COX4, only in p53-expressing HCT116 tumors.

In the same tumor tissue, the expression levels of markers associated with angiogenesis (VEGF1) and migration (MMP-9 and E-CAD) were also evaluated. When compared to vehicle, SLMP53-1 treatment decreased the expression levels of MMP-9 and VEGF1, while increasing the levels of E-CAD in p53^+/+^, but not in p53^−/−^, HCT116 tumors (Figure 2A,B).

Collectively, likewise in vitro, SLMP53-1 displayed the in vivo potential to antagonize the Warburg effect, also inhibiting angiogenesis and modulating key signatures of ECM remodeling and EMT processes, in vivo in a p53-dependent manner.

### 2.3. SLMP53-1 Synergizes with Dichloroacetic Acid (DCA), Enhancing Its Antitumor Efficacy in Cancer Cells

**Figure 3:**
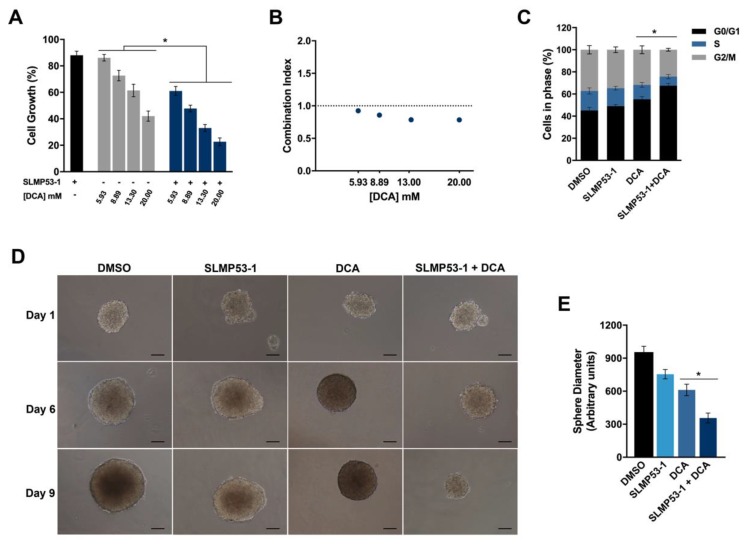
SLMP53-1 sensitizes cancer cells to DCA. (**A**) HCT116 cells were treated with 5.93–20 mM DCA alone and in combination with 9 μM SLMP53-1; cell proliferation was determined after 48 h treatment; growth obtained with solvent (DMSO) was set as 100%. Data are mean ± SEM of six independent experiments. Values significantly different from DCA alone (* *p* < 0.05, two-way ANOVA followed by Sidak’s test). (**B**) Combination index (CI) values calculated by using the CompuSyn software for each combined treatment. CI < 1, synergy; 1 < CI < 1.1, additive effect; CI > 1.1, antagonism. CI values were calculated by using a mean value effect of six independent experiments. (**C**) Effect of 13.3 mM DCA alone and in combination with 9 μM SLMP53-1 in cell cycle of HCT116 cells, after 48 h treatment. Data are mean ± SEM of three independent experiments. Values significantly different from DCA alone (* *p* < 0.05, two-way ANOVA followed by Dunnett’s test). (**D**) Effect of 20 μM SLMP53-1 alone and in combination with 12 mM DCA on three-day-old HCT116 colonosphere, for up to nine days of treatment. Images are representative of three independent experiments (scale bar = 50 μm; magnification = ×100). (**E**) Determination of spheroid diameter at the end of treatment; data represent mean ± SEM of three independent experiments. Values significantly different from DCA alone (* *p* < 0.05, one-way ANOVA followed by Dunnett’s test).

We also intended to assess the ability of SLMP53-1 to enhance the antitumor activity of the well-established metabolic regulator DCA, in HCT116 cells. For that, cancer cells were treated with SLMP53-1 at 9 µM (concentration with no significant anti-proliferative effect on HCT116 cells [24]) and/or with 5.93–20 mM of DCA. The results showed a significant enhancement of the growth inhibitory effect of DCA by SLMP53-1 (Figure 3A). A multiple-drug-effect analysis was performed with the calculation of the combination index (CI) for each combination. As depicted in Figure 3B, synergistic effects between SLMP53-1 and DCA could be observed for the four concentrations tested (CI ˂ 1). Moreover, for the combination of 13.3 mM DCA with 9 μM SLMP53-1, it was observed that the synergic effect was associated with an increase of G0/G1-phase cell cycle arrest, when compared to DCA only-treated cells (Figure 3C). Accordingly, in a 3D HCT116 spheroid model, we could corroborate that the growth inhibitory activity of DCA was visibly potentiated by SLMP53-1 at 20 µM (concentration with no significant effect on HCT116 colonosphere growth), after nine days treatment (Figure 3D).

## 3. Discussion

The high sensitivity of cancer cells to the counteraction of Warburg effect has been considered a valuable target in anticancer therapy; in particular, activation of p53, a major regulator of glucose metabolism, can conduct to a robust inhibition of energy production in cancer cells and subsequently to their efficient elimination. In fact, among the wide set of genes regulated by p53, important regulators of glucose metabolism are included, which have central roles in maintaining mitochondrial health, increasing mitochondrial respiration, and lowering glycolysis (reviewed in [26]).

In earlier studies, SLMP53-1 was identified as a new activator of wt- and mutp53, with encouraging in vitro and in vivo p53-dependent antitumor activity [24,27]. Data from SwissADME analysis [25] also showed that SLMP53-1 followed criteria for drug-likeness, gastrointestinal adsorption, lipophilicity, and solubility (Supplementary Tables S1–S7). This evidence led us to further investigate the potential of SLMP53-1 as an anticancer drug candidate, particularly through an in-depth analysis of its impact on glucose metabolism, OXPHOS and glycolysis. In fact, it was shown that SLMP53-1 triggered a p53-dependent mitochondrial apoptotic cell death in cancer cells, involving upregulation of BAX and PUMA expression levels (considered a consequence of the stimulation of both pro-oxidant and mitochondrial p53 functions [28]), mitochondrial membrane potential dissipation, p53 and BAX mitochondrial translocation, cytochrome *c* release, and p53-dependent ROS generation [24]. ROS are a toxic by-product of OXPHOS, playing a major role in the progressive accumulation of damages in cancer cells and in their efficient elimination through apoptosis [29].

In this work, the results in cancer cells and xenograft human tumor tissues unveiled that SLMP53-1 potentially stimulated OXPHOS through upregulation of SCO2, which in turn is involved in ROS accumulation and apoptotic cell death. In fact, some authors have reported that SCO2 upregulation provides an alternative pathway of p53-dependent apoptosis [30]. SLMP53-1 also caused in vitro and in vivo upregulation of COX4, a nuclear-encoded subunit of mitochondrial complex IV, and increased the levels of OXPHOS mitochondrial complexes in cancer cells, which further reinforced the stimulation of OXPHOS by the compound. Additionally, SLMP53-1 inhibited glycolysis, with downregulation of several glycolytic intervenient, including glucose transporters (GLUT1) and enzymes (HK2 and PFKFB3), which are direct p53 targets, in cancer cells and xenograft tumor tissue. Thus, p53 activation by SLMP53-1 ablates the first step of glycolysis, namely glucose uptake, primary phosphorylation of glucose molecules, and the rate-limiting step of glycolysis, the conversion of fructose-6-phosphate to fructose-1,6-biphosphate. Notably, these effects of SLMP53-1 on OXPHOS and glycolysis were shown to be dependent on p53 in xenograft tumor tissue. Notably, consistently with in vivo data, it was also observed in vitro that SLMP53-1 did not interfere with the expression levels of glycolytic markers such as HK2 and GLUT1, in HCT116 p53^−/−^ cells (Supplementary Figure S2).

As previously mentioned, elevated lactate levels are correlated with poor prognosis and decreased overall survival in cancer patients [31]. In fact, high lactate production promotes the metastatic disease, which largely remains the main cause of cancer-associated morbidity and mortality [32]. Herein, it is shown that SLMP53-1 also significantly reduced the extracellular lactate levels in cancer cells. Accordingly, a significant downregulation of MCT4 expression levels, a plasma membrane lactate transporter, was observed in SLMP53-1-treated tumor tissue. The MCT4 expression is not directly regulated by p53. Instead, its expression is induced via HIF-1 [33]. Indeed, in response to the hypoxic conditions in tumors, activated HIF-1 upregulates transporters and enzymes of the glycolysis pathway, while reducing mitochondrial respiration [34]. However, p53 makes part of a triad with *c*-Myc and HIF-1, which is responsible for the transcription of multiple genes that tightly regulate cell metabolism through feedback mechanisms [34]. Activation of p53 keeps the levels of HIF-1 low, with downregulation of transporters regulated by HIF-1, including MCT4 [34]. Interestingly, MCT4 is overexpressed in several cancers, which is correlated with cancer progression, infiltration, and angiogenesis [35]. MCT4 is therefore recognized as a marker of poor prognosis [36]. As such, several inhibitors of MCTs, including MCT4, have been evaluated as promising anticancer therapeutic options. For example, the MCT1/4 inhibitor syrosingopine [37] and the MCT1 inhibitor AZD3965 [38] have shown promising preclinical anticancer activity. In fact, AZD3965 is currently under phase I trial for refractory and advanced solid tumors and lymphoma (NCT07197595). Herein, we also showed that SLMP53-1 modulated in vitro and in vivo key signatures of ECM remodeling and EMT processes, increasing E-CAD, while decreasing MMP-9. Importantly, this effect showed to be dependent on p53 in xenograft tumor tissue. In fact, this is in accordance with our previous work, in which low doses of SLMP53-1 significantly inhibited the migration of colon cancer cells (wound healing assay) [23].

In this work, the anti-angiogenic activity of SLMP53-1 was also confirmed. In fact, a pronounced reduction of VEGF production by SLMP53-1 was observed in p53-expressing cancer cells. Indeed, it was reported that p53 indirectly suppresses VEGF expression by repressing transcription factors like SP1 [39] and E2F [40,41]. Interestingly, SLMP53-1 also inhibited the VEGF expression levels in endothelial cells, an effect associated with reduction in cell tube formation. Further, although this negative effect of SLMP53-1 on VEGF production could be also observed in p53-expressing tumor tissue, it was depleted in p53-null tumor tissue. These results evidenced that the anti-angiogenic activity of SLMP53-1 was highly dependent on tumor environment, particularly on its p53 status. In fact, the high VEGF expression levels by p53-null cancer cells seem to counteract the inhibitory effect of SLMP53-1 on VEGF production by endothelial cells. Given the fact that angiogenesis is an essential event in tumor dissemination and metastases formation [42], these results further reinforced the p53-dependent anti-metastatic activity of SLMP53-1.

Our data further corroborated the combination of drugs targeting glucose metabolism with p53-activating agents as an efficient therapeutic approach to counteract resistances and undesirable toxicity commonly observed in cancer therapy. Indeed, SLMP53-1 was shown to synergize with DCA both in 2D and 3D cancer cell models. DCA is a well-established inhibitor of the mitochondrial pyruvate dehydrogenase kinase (PDK) [43,44] that was shown to reverse the Warburg effect [45]. DCA binds to PDK and attenuates the inhibition of PDH activity, which shifts metabolism from glycolysis to OXPHOS, with subsequent increase of ROS production, decrease of mitochondrial membrane potential, efflux of pro-apoptotic mediators from the mitochondria, and induction of mitochondria-dependent apoptosis [45]. Interestingly, some authors have recently shown that DCA restored colorectal cancer chemosensitivity through the p53/miR-149-3p/PDK2-mediated glucose metabolic pathway [46]. Accordingly, it was also reported that the antitumor activity of DCA was dependent on a functional p53 status, exhibiting synergistic growth inhibitory effects in combination with the p53 activator Nutlin-3 [47].

As a whole, this work further reinforces the great potential of SLMP53-1, as single or combined anticancer drug, by targeting major hallmarks of cancer growth and dissemination.

## 4. Materials and Methods

### 4.1. Compounds and Reagents

(*S*)-tryptophanol-derived oxazoloisoindolinone (SLMP53-1) was synthesized by using the protocol previously described in [24]. For all experiments, SLMP53-1 was dissolved in dimethyl sulfoxide (DMSO) from Sigma-Aldrich (Sintra, Portugal). Dichloroacetic acid (DCA) from Sigma-Aldrich was dissolved in water. Primary antibodies used in Western blot and/or immunohistochemistry were as follows: anti-α-Tubulin (Sigma-Aldrich, T9026), anti-COX4 (F-8; Santa Cruz Biotechnology, sc-376731), anti-E-Cadherin (G-10; Santa Cruz Biotechnology, sc-8426), anti-GAPDH (6C5; Santa Cruz Biotechnology, sc-32233), anti-GLUT1 (Abcam, ab652), anti-HK2 (3D3; Merck Millipore, MABN702), anti-MCT4 (H-90; Santa Cruz Biotechnology, sc-50329), anti-MMP9 (2C3; Santa Cruz Biotechnology, sc-21733), anti-N-Cadherin (13A9; Santa Cruz Biotechnology, sc-59987), anti-PFKFB3 (ThermoScientific, PA5-21931), anti-SCO2 (ProteinTech, 21223-1-AP), total OXPHOS antibody cocktail (Abcam, ab110413), and anti-VEGF1 (ThermoScientific, MA1-16629). Secondary antibodies used in Western blot were as follows: anti-mouse (Abcam, ab6789) and anti-rabbit (Santa Cruz Biotechnology, sc-2004) horseradish peroxidase-conjugated.

### 4.2. Human Cell Lines and Growth Conditions

Human colon adenocarcinoma HCT116 cell lines expressing wt p53 were provided by B. Vogelstein (The Johns Hopkins Kimmel Cancer Center, Baltimore, MD, USA) and were routinely cultured in RPMI 1640 medium with UltraGlutamine from Lonza (VWR, Carnaxide, Portugal) supplemented with 10% fetal bovine serum (FBS) from Gibco (Alfagene, Lisboa, Portugal). Dermal microvascular endothelial HMVEC-D cells (Lonza, VWR) were cultured in endothelial cell growth basal medium supplemented with SingleQuots™ Kit (Lonza, VWR). Both cells were maintained at 37 °C in a humidified atmosphere of 5% CO_2_. Cells were routinely tested for mycoplasma infection by using the MycoAlert™ PLUS mycoplasma detection kit (Lonza, VWR).

### 4.3. Immunohistochemistry (IHC)

Tumor tissues from p53^+/+^ and p53^−/−^ colon HCT116 cancer xenografts treated with 50 mg/kg SLMP53-1 or vehicle by intraperitoneal injection twice a week, for a total of five administrations, were obtained in previous study [24]. Tissues were fixed in 10% formalin, embedded in paraffin, sectioned at 4 μm, and stained with hematoxylin and eosin (H&E) or antibodies, as described in [24]. Briefly, antigen retrieval was performed by boiling the sections for 20 min in citrate (pH 6.0) or EDTA (pH 8.0) buffer. Antibodies used are listed in Section 4.1. Immunostaining was performed by using the UltraVision Quanto Detection System HRP DAB Kit, from Lab Vision Thermo Scientific (Grupo Taper SA, Sintra, Portugal), according to the manufacturer’s instructions. Evaluation of DAB (3,3′-diaminobenzidine) intensity and quantification of marked cells were performed by using Image J software (Laboratory for optical and computational instrumentation, University of Wisconsin-Madison, USA). Images were obtained by using an Eclipse E400 fluorescence microscope (Nikon) with ×200 magnification, with a Digital Sight camera system (Nikon DS-5Mc) and software Nikon ACT-2U (Izasa Carnaxide, Portugal).

### 4.4. Western Blot

To evaluate the expression levels of HK2, GLUT1, PFKFB3, SCO2, COX4, E-CAD, N-CAD, and VEGF1, HCT116 cells were seeded in 6-well plates at 1.5 × 10^5^ cells/well density for 24 h. Cancer cells were lysed and protein fractions were analyzed as described in [24]. Antibodies used are described in Section 4.1.

### 4.5. Measurement of Extracellular Lactate

The levels of lactate exported by HCT116 were determined by using the Lactate-Glo^TM^ assay kit (Promega, J5021, VWR) according to the manufacturer’s instructions. Briefly, 5.0 × 10^3^ HCT116 cells/well were seeded in 96-well plates, followed by treatment with SLMP53-1 or solvent for 24 h. The culture medium was then collected and diluted in phosphate saline buffer. After that, 50 μL was transferred to a 96-well assay plate, and 50 μL of Lactate Detection Reagent was added. After 60 min at room temperature, luminescence was read by using the Bio-Tek Synergy HT plate reader (Izasa).

### 4.6. Angiogenesis Assay

Endothelial tube formation was evaluated by using the in vitro Angiogenesis Assay Kit (Millipore, VWR) according to the manufacturer’s instructions. Briefly, 3 × 10^4^ HMVEC-D cells/well were seeded in 24-well plates coated with ECMatrix with SLMP53-1 or solvent for 12 h. Cells were photographed by using the inverted Nikon TE 2000-U microscope at ×100 magnification, with a DXM1200F digital camera and NIS-Elements microscope imaging software (VWR).

### 4.7. Generation of Colon Cancer Spheroids

HCT116 cells were resuspended in serum-free stem cell culture media consisting of DMEM/F12 supplemented with 10 ng·mL^−1^ of bFGF, 20 ng·mL^−1^ of EGF from Biotechne (Citomed Lda, Lisboa, Portugal), 1 × B27 from Life Technologies (Porto, Portugal), and 5 μg·mL^−1^ of insulin (Sigma-Aldrich). HCT116 cells were plated in 24-well ultra-low attachment plates (one spheroid per well; Corning Inc., Sigma-Aldrich), at a density of 1 × 10^3^ cells/well [48]. To assess the synergistic effect of SLMP53-1 with DCA in spheroids development, colonospheres were allowed to form for 3 days, followed by treatment with 20 μM SLMP53-1 and/or 12 mM DCA, for an additional 9 days. During this period of time, new medium with the drugs (or DMSO only) was added to the wells each three days. Spheroids were photographed by using an inverted Nikon TE 2000-U microscope at ×100 magnification, with a DXM1200F digital camera and NIS-Elements microscope imaging software (VWR). Determination of spheroids diameter was performed by using Image J software.

### 4.8. Combination Therapy Assays

To assess the synergistic effect of SLMP53-1 with DCA, HCT116 cells were treated with 9 μM SLMP53-1 and/or increasing concentrations of DCA (5.93–20 mM) for 48 h. The effect of combined treatments on cell proliferation was analyzed by SRB assay. For each combination, the combination index (CI) values were calculated by using the CompuSyn Software version 1.0 (ComboSyn, Inc., Paramus, NJ, USA), according to the following equation: CI = (D)_1_/(D_x_)_1_ + (D)_2_/(D_x_)_2_, where the numerators (D)_1_ and (D)_2_ are the concentrations of each drug in the combination (D)_1_ + (D)_2_ that inhibit x%, and the denominators (D_x_)_1_ and (D_x_)_2_ are the concentrations of drug one and two alone that inhibit x%; DRI measures how much the dose of a drug may be reduced in synergistic combination compared to the dose of each drug alone; CI values < 1, 1 < CI < 1.1, and > 1.1 indicate synergistic, additive and antagonistic effects, respectively [49].

### 4.9. Cell Cycle Analysis

The analyses were performed basically as described by [24]. Briefly, HCT116 cells were seeded in six-well plates at a density of 1.5 × 10^5^ cells/well for 24 h, followed by treatment with SLMP53-1, DCA, or both, for an additional 48 h. Cells were then stained with propidium iodide (Sigma-Aldrich) and analyzed by flow cytometry; cell cycle phases were identified and quantified by using the FlowJo X 10.0.7 Software (Treestar, Ashland, OR, USA). The AccuriTM C6 flow cytometer and the BD Accuri C6 software (BD Biosciences, Enzifarma, Porto, Portugal) were used.

### 4.10. Statistics

Data were statistically analyzed by using GraphPad Prism version 7.0 (San Diego, CA, USA). Different statistical tests were used, accordingly, to the dataset; *p* values < 0.05 were considered statistically significant.

## Supplementary Materials

Supplementary materials can be found at https://www.mdpi.com/1422-0067/21/2/596/s1.
