# Supplementary material for: SLMP53-1 Inhibits Tumor Cell Growth through Regulation of Glucose Metabolism and Angiogenesis in a P53-Dependent Manner
Source: Int J Mol Sci. 2020 Jan 17;21(2):596. doi: 10.3390/ijms21020596 (PMC7013701; doi:10.3390/ijms21020596)
Supplement: Supplementary file 1 [file ijms-21-00596-s001.pdf]

SLMP53-1 Inhibits Tumor Cell Growth through Regulation of Glucose Metabolism and Angiogenesis in a P53-Dependent Manner

Helena Ramos, Juliana Calheiros, Joana Almeida, Valentina Barcherini, Sónia Santos, Alexandra T. P. Carvalho, Maria M.M. Santos and Lucília Saraiva

Physicochemical and pharmacokinetic parameters of SLMP53-1 calculated using SwissADME (1): The resemblance of the SLMP53-1 compound to other drugs was evaluated with the Ghose, Egan, Veber and Muegee filters. The SLMP53-1 molecule met all the criteria set by those filters. Table S1 provides detailed data regarding the Ghose qualification criteria, which contain physicochemical properties like the molar refractivity (MR; between 40 and 130), molecular weight (MW; between 160 and 480) and number of atoms (20 to 70 atoms). SLMP53-1 also obeyed the Veber model criteria, meaning that it is more likely to have better oral bioavailability (10 or less rotary titers and a polar surface area, PSA, equal to or fewer than 140 Å2 with 12 or fewer hydrogen binding donors and acceptors). Finally, it obeyed the Muegge and Egan filters (Table S2). The Muegge model is an independent database capable of discriminating between drug and chemical substances similar to drugs. The Egan filter is based on the PSA and the AlogP98v, providing a robust prediction of drug absorption. SLMP53-1 was also evaluated against models that exclude molecules that are likely to fail biological assays. The analysis showed no warnings from Pains Alert. In addition, the Brenk selection model and the Leadlikeness criteria also proved to be non-infringing (Table S3). Pharmacokinetic evaluation by SwissADME also estimated that SLMP53-1 has high gastrointestinal absorption, is capable to penetrate the blood brain barrier (BBB), and has an inhibitory function on cytochrome p450 isoenzymes CYP1A2 and CYP2D6 (Table S4). Water solubility was evaluated according to 3 models: the ESOL model, an adapted model from Ali *et* al, and a model developed by SILICOS-IT. The molecule was evaluated as soluble, soluble and moderately soluble respectively. All values given are the decimal logarithm of molar solubility in water (*log S*) (Tables S5 and S6). The lipophilicity of the molecule was further evaluated with Consensus *Log P*, being in the average value of all *Log P* calculated with various lipophilicity criteria (Table S7).

Table 1. Physicochemical properties.

MOLECULE	Canonical SMILES	Formula	MW	#Heavy atoms	#Aromatic heavy atoms	Fraction Csp3	#Rotatable bonds	#H-bond acceptors	#H-bond donors	MR	TPSA
SLMP53-1	O=C1C2C=CC=CC2[C@@]2(N1[C@H](CO2)Cc1c[nH]c2c1cccc2)C	C20H20N2O2	320.39	24	9	0.35	2	2	1	96.99	45.33

(MW; molecular weight, MR; molar refractivity, TPSA; total polar surface area).

Table 2. SwissADME shows that SLMP53-1 do not violate any of the drug-likeness criteria.

MOLECULE	Canonical SMILES	Lipinski #violations	Ghose #violations	Veber #violations	Egan #violations	Muegge #violations
SLMP53-1	O=C1C2C=CC=CC2[C@@]2(N1[C@H](CO2)Cc1c[nH]c2c1cccc2)C	0	0	0	0	0

Table 3. Medicinal chemistry evaluation of SLMP53-1.

MOLECULE	Canonical SMILES	PAINS #alerts	Brenk #alerts	Leadlikeness #violations	Synthetic Accessibility
SLMP53-1	O=C1C2C=CC=CC2[C@@]2(N1[C@H](CO2)Cc1c[nH]c2c1cccc2)C	0	0	0	4.49

Table 4. Pharmacokinetic evaluation of the SLMP53-1.

MOLECULE	Canonical SMILES	GI absorption	BBB permeant	Pgp substrate	CYP1A2 inhibitor	CYP2C19 inhibitor	CYP2C9 inhibitor	CYP2D6 inhibitor	CYP3A4 inhibitor
SLMP53-1	O=C1C2C=CC=CC2[C@@]2(N1[C@H](CO2)Cc1c[nH]c2c1cccc2)C	High	Yes	No	Yes	No	No	Yes	No

(GI; gastro-intestinal absorption, BBB; blood brain barrier, nCYP; Cytochromes, P-gp; P-glycoprotein).

Table 5. Water solubility evaluation of SLMP53-1 (ESOL and Ali Ali *et al*. models).

MOLECULE	Canonical SMILES	ESOL Log S	ESOL Solubility (mg/ml)	ESOL Solubility (mol/l)	ESOL Class	Ali Log S	Ali Solubility (mg/ml)	Ali Solubility (mol/l)	Ali Class
SLMP53-1	O=C1C2C=CC=CC2[C@@]2(N1[C@H](CO2)Cc1c[nH]c2c1cccc2)C	-3.88	4.22e-02	1.32e-04	Soluble	-3.65	7.22e-02	2.25e-04	Soluble

Table 6. Water solubility evaluation of SLMP53-1 (SILICOS-IT model).

MOLECULE	Canonical SMILES	Silicos-IT LogSw	Silicos-IT Solubility (mg/ml)	Silicos-IT Solubility (mol/l)	Silicos-IT class
SLMP53-1	O=C1C2C=CC=CC2[C@@]2(N1[C@H](CO2)Cc1c[nH]c2c1cccc2)C	-4.15	2.27e-02	7.08e-05	Moderately soluble

Table 7. Lipophilicity evaluation of SLMP53-1.

MOLECULE	Canonical SMILES	iLOGP	XLOGP3	WLOGP	MLOGP	Silicos-IT Log P	Consensus Log P
SLMP53-1	O=C1C2C=CC=CC2[C@@]2(N1[C@H](CO2)Cc1c[nH]c2c1cccc2)C	2.50	3.03	2.65	2.66	2.74	2.71

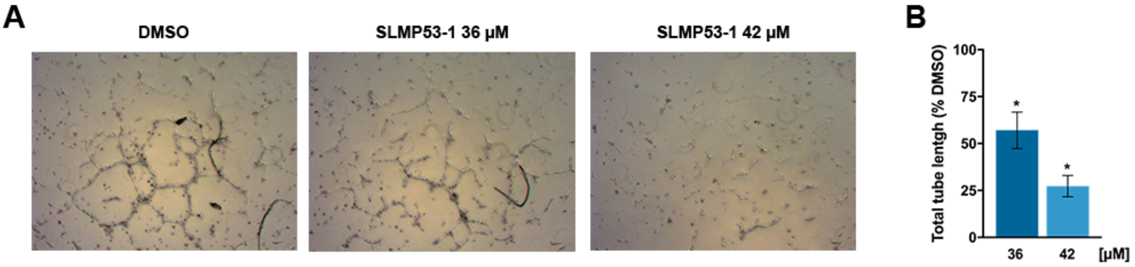

Figure S1. Anti-angiogenic effect of SLMP53-1, in HMVEC-D cells, after 12 h treatment, using the endothelial tube formation assay. (A) Representative images are shown (magnification = ×40). (B) Quantification of total endothelial tube length in five randomly selected microscopic fields; percentages are relative to solvent (DMSO) and correspond to mean ± SEM of three independent experiments. Values significantly different from DMSO (**p* < 0.05; one-way ANOVA with Dunnett's multiple comparison test).

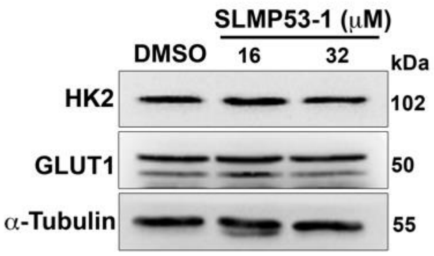

Figure S2. Expression levels of HK2 and GLUT1, after 24 h treatment with SLMP53-1, in HCT116 $p53^{-/-}$ cancer cells. Immunoblots are representative of two independent experiments; α-tubulin was used as a loading control.

References

1. Daina A, Michielin O, Zoete V. SwissADME: a free web tool to evaluate pharmacokinetics, drug-likeness and medicinal chemistry friendliness of small molecules. Sci Rep. 2017;7:42717.
